# Procalcitonin and C-reactive protein in urinary tract infection diagnosis

**DOI:** 10.1186/1471-2490-14-45

**Published:** 2014-05-30

**Authors:** Rui-Ying Xu, Hua-Wei Liu, Ji-Ling Liu, Jun-Hua Dong

**Affiliations:** 1 Department of Pediatrics, Qilu Hospital of Shan Dong University, Jinan 250012, China

**Keywords:** Urinary tract infections, Acute pyelonephritis, Receiver operating characteristic curve, Procalcitonin

## Abstract

**Background:**

Urinary infections are a common type of pediatric disease, and their treatment and prognosis are closely correlated with infection location. Common clinical manifestations and laboratory tests are insufficient to differentiate between acute pyelonephritis and lower urinary tract infection. This study was conducted to explore a diagnostic method for upper and lower urinary tract infection differentiation.

**Methods:**

The diagnostic values of procalcitonin (PCT) and C-reactive protein (CRP) were analyzed using the receiver operating characteristic curve method for upper and lower urinary tract infection differentiation. PCT was determined using chemiluminescent immunoassay.

**Results:**

The PCT and CRP values in children with acute pyelonephritis were significantly higher than those in children with lower urinary tract infection (3.90 ± 3.51 ng/ml and 68.17 ± 39.42 mg/l vs. 0.48 ± 0.39 ng/ml and 21.39 ± 14.92 mg/l). The PCT values were correlated with the degree of renal involvement, whereas the CRP values failed to show such a significant correlation. PCT had a sensitivity of 90.47% and a specificity of 88% in predicting nephropathia, whereas CRP had sensitivity of 85.71% and a specificity of 48%.

**Conclusions:**

Both PCT and CRP can be used for upper and lower urinary tract infection differentiation, but PCT has higher sensitivity and specificity in predicting pyelonephritis than CRP. PCT showed better results than CRP. PCT values were also correlated with the degree of renal involvement.

## Background

Urinary infections are a common pediatric disease and their treatment and prognosis are closely correlated with infection location. Common clinical manifestations and laboratory indices are insufficient for acute pyelonephritis and lower urinary tract infection (UTI) differentiation. Differentiating between these diseases is particularly difficult for infants and children, but is necessary because pyelonephritis poses a risk of renal parenchyma involvement, which can lead to renal scar formation, as well as high blood pressure and end stage renal failure in adults [1-4]. Smellie et al. conducted a retrospective analysis of 52 patients with neodevelopment or progressive renal scars and revealed that 50 of these patients had a medical history of urinary infection diagnosis or treatment delay [5]. Therefore, finding a technically easy and practical method for differentiating between upper and lower UTIs is urgently required.

As of this writing, ^99m^Tc-dimercaptosuccinic acid (DMSA) scintigraphy is a commonly adopted method for diagnosing severity degrees of renal involvement and pyelonephritis. However, this method is costly and is radioactive to sick children [6,7]. Procalcitonin (PCT) is a type of hormonal activity-free calcitonin precursor protein that can serve as an early diagnosis index of serious bacterial infections and septicemia; PCT is also correlated with the severity of bacterial infections [8-10]. PCT is a satisfactory predictor of renal parenchymal involvement in acute and late renal scars [11-13].

We retrospectively analyzed the diagnostic value of PCT in differentiating upper and lower UTIs, and the serum PCT level was determined and compared with C-reactive protein (CRP) and peripheral blood leukocyte count; the results were subsequently analyzed using the receiver operating characteristic (ROC) method [14,15].

## Methods

### Clinical data

A total of 46 patients with suspected acute pyelonephritis (APN) were enrolled in the study from December 1999 to April 2002. Twenty-eight females and 18 males were included, and their ages ranged from 2 months old to 14 years old. Inclusion criteria were as follows: fever (≥38.5°C, axillary), pyuria (≥10 white blood cells per high-power field on a spun urine), and positive urine culture (≥100,000 colonies/ml of a single organism, clean catch). Exclusion criteria included the presence of renal calculi, obstructive uropathy, and a neurogenic bladder.

We performed a retrospective analysis of 46 admitted patients, who underwent a DMSA renal scan for suspected APN within 5 d of admission.

APN was confirmed using radioactive nuclide ^99m^Tc-DMSA scanning. Diagnostic criteria were based on literature [16]. APN is indicated when radioactive renal parenchyma distributional sparse or loss areas are present and accompanied with swelling or a normal kidney profile. Renal scar formation is diagnosed when the kidney volume decreases (manifested by cortex attenuation, renal morphologic abnormality, or profile shrinkage) with wedge-shaped defects. Renal involvement was graded as follows: renal injury <25% was considered mild; renal injury between 25% and 50% was considered moderate; and renal injury >50% was considered serious. Lower UTI was diagnosed by normal DMSA scanning. If the first DMSA findings were abnormal, another analysis was performed six months later. All patients underwent renal ultrasonography within 48 h of admission, and eight recurrent patients underwent voiding cystourethrography within 7 d of admission.

### Experimental methods

The patients were divided into APN and lower UTI groups and treated as follows: two weeks of antibiotic treatment for APN patients, 7 d of antibiotic treatment for Lower UTI. PCT was determined using chemiluminescent immunoassay, and CRP was scored using nephrometry scoring. PCT, CRP, and white blood cell (WBC) count were determined within 24 h of admission and 24 h of therapy.

### Statistical analysis

All data were normally distributed and were presented as mean ± standard error ($\overset{¯}{x}\pm s$). *T*-tests were performed to compare the means between groups. Cut points were selected and then graded according to normal, basically normal, susceptible, basically abnormal, and abnormal classifications. Se and Sp of each point were calculated. Taking Se as the ordinate, which represented true positive rate, and (1-Sp) as the abscissa, which represented false positive rate. ROC curves were drawn by the SPSS 10.0 software to calculate the area under curve and standard errors. The cut-off value was selected depending on (Sp + Se)_max_. Values of different indices were also compared.

This study was conducted in accordance with the declaration of Helsinki and with approval from the Ethics Committee of Qilu Hospital of Shan Dong University. Written informed consent was obtained from all parent or guardian of participants.

## Results

### Clinical data

A total of 46 children with urinary infection who received treatment between December 1999 and April 2002 were enrolled. Among the patients, 18 were males and 28 were females. Their ages ranged from 2 months old to 14 years old: six males and two females were <1 year old, seven males and 11 females were between 1 year old and 3 years old, and five males and 15 females were ≥3 years old. A total of 38 patients had primary urinary infection, and eight had recurrent infection. Their courses of disease ranged from 3 d to 1 yr. These patients did not receive antibiotic treatment within half a month before hospitalization. A total of 40 patients had body temperatures between 38.5°C and 40.0°C. Twenty-eight patients presented UTI symptoms, such as frequent micturition, urgent micturition, odynuria, and crying while urinating. Twelve patients had lumbago and percussion pain on kidney region. Four patients had macroscopic hematuria. Eight patients, six of whom were less than 2 years old, presented non-specific signs and symptoms, such as emesis, diarrhea, abdominal pain, poor disposition and appetite, icterus, and irritability. All patients had WBC count >10/HP, 12 had red blood cell count of >5/HP, and 11 had urine protein between + and ++, according to routine urine examination. Urine culture outcomes of all patients were positive.

### Radioactive nuclide scanning

Twenty-one out of the 46 patients were diagnosed with APN. Among these patients, two had non-obstructive hydronephrosis, one presented renal scar formation, and two presented vesico-ureteral reflux.

### PCT and CRP

The serum PCT and CRP levels of the APN group were significantly higher than those of the lower UTI group (3.90 ± 3.51 ng/ml and 68.17 ± 39.42 mg/l vs. 0.48 ± 0.39 ng/ml and 21.39 ± 14.92 mg/l, *P* < 0.01, Table 1). Correlation analysis demonstrated that PCT and CRP were in a significantly positive correlation with a correlation coefficient of 0.729 (*P* < 0.01).

**Table 1:** **Comparisons of the laboratory outcomes between groups (**$\overset{¯}{x}\pm s$)

**Group**	**PCT (ρ/ng · ml**^**-1**^**)**	**CRP (ρ/mg · l**^**-1**^**)**	**WBC number (/mm**^**3**^**)**
Lower urine tract infection	0.48 ± 0.39	21.39 ± 14.92	14068 ± 6870
Acute pyelonephritis	3.90 ± 3.51	68.17 ± 39.42	15882 ± 7350
*p* value	< 0.01	< 0.01	> 0.05

### Curve analysis

**Figure 1:**
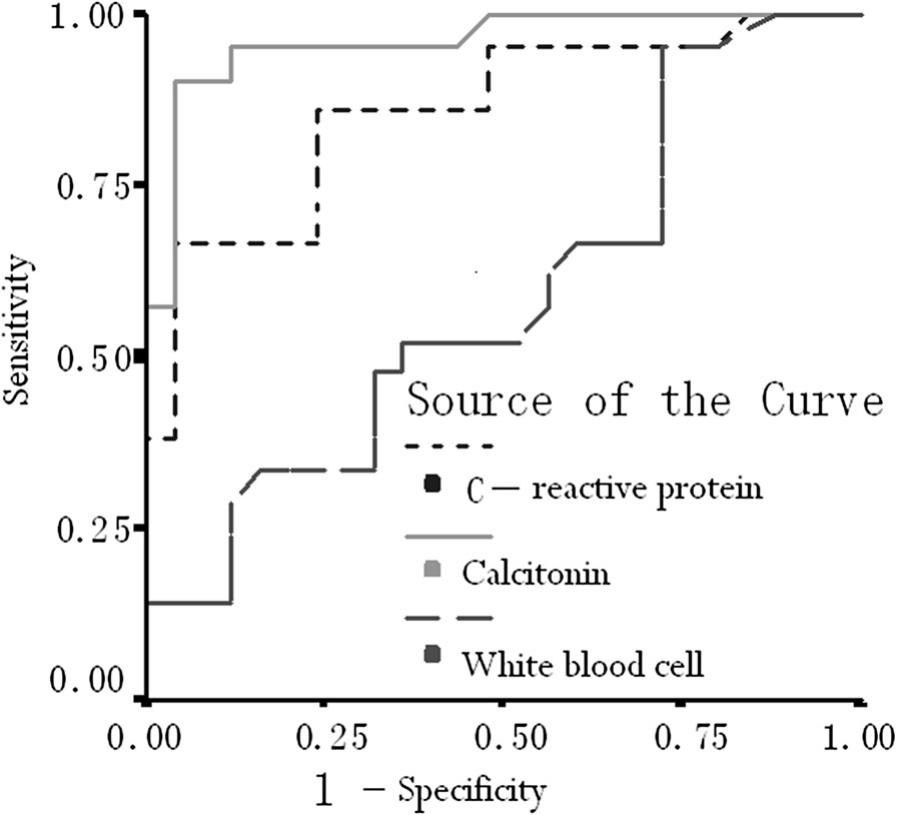
Comparison of the ROC curves of PCT, CRP and WBC.

As shown in Figure 1, the areas under the PCT, CRP, and WBC curves were 0.958, 0.858, and 0.588, respectively. Group comparison analysis showed no significant difference between the areas under the PCT and CRP curves (*P* > 0.05), whereas the areas under these curves were significantly larger than that under the WBC curve (*P* < 0.01). PCT and CRP are highly accurate in diagnosing APN.

### Diagnostic values

The diagnostic values of PCT, CRP, and WBC were 1.0 ng/ml, 20 mg/l, and 15,000/mm^3^, respectively. The sensitivity and specificity of PCT in predicting nephropathia were 90.47% and 88%, whereas those of CRP were 85.71% and 48%, respectively. PCT had the highest sensitivity and specificity in diagnosing APN among the three methods. The results are summarized in Table 2.

**Table 2:** Comparisons of the diagnostic values of PCR, CRP, and WBC for acute pyelonephritis (%)

**Index**	**Diagnostic reference value**	**Sensitivity**	**Specificity**	**Accuracy**	**Positive predictive value**	**Negative predictive value**
PCT	1 ng/ml	90.47	88	89	87	95
CRP	20 mg/l	85.71	48	86.9	58	80
WBC	15000/mm^3^	57	44	71	46	55

### Renal involvement degrees

The PCT level in children with serious renal involvement (8.60 ± 2.80 ng/ml) was noticeably higher than that in children with mild and moderate renal involvement (2.02 ± 1.24 ng/ml; *P* < 0.01), whereas the WBC counts and serum CRP levels among children with different renal involvement degrees did not show significant differences (*P* > 0.05). High PCT value indicates a serious degree of renal involvement. The results are summarized in Table 3.

**Table 3:** Comparisons between laboratory outcomes and renal involvement degrees

**Index**	**Mild and moderate degrees (n = 15; DMSA scanning)**	**Serious degree (n = 6; DMSA scanning)**	***p *** **values**
PCT (ρ/ng · ml^-1^)	2.02 ± 1.24	8.60 ± 2.80	< 0.01
CRP (ρ/mg · l^-1^)	62.0 ± 42.83	82.02 ± 28.56	> 0.05
WBC (cells/mm^3^)	14990 ± 2611	15980 ± 3220	> 0.05

### PCT and CRP outcome analysis

The pre- and post-treatment PCT levels were 3.90 ± 3.51 and 1.78 ± 2.07 ng/ml, respectively, and the pre- and post-treatment CRP levels were 68.17 ± 39.42 and 26.13 ± 15.14 mg/l. Great differences in the PCT and CRP were observed before and after treatment (*P* < 0.05). Follow-up DMSA scanning at six months after treatment showed that 15 children completely recovered, three showed great improvement, two showed little improvement, and one failed to show noticeable improvement. The child who failed to show noticeable improvement had renal scars with vesico-ureteral reflux before the hospitalization, whereas all other patients had no new scars. The serum PCT levels (>10 ng/ml) in three patients before treatment were still higher than 5 ng/ml after treatment.

## Discussion and conclusion

Urinary infections are a common type of pediatric disease, and their treatment and prognosis are closely associated with infection position. Differentiating APN from lower UTI is difficult based on common clinical manifestations and laboratory indices, and thus, an easier and more practical method is necessary. ^99m^Tc-DMSA scintigraphy is currently a commonly adopted method for the diagnosis of renal involvement degree and pyelonephritis; this method is costly and radioactive to children [6,7].

PCT is a hormonal activity-free calcitonin precursor protein [17]. Studies have proven that PCT serves as an early diagnosis index for bacterial infections and septicemia, and that PCT is also correlated with the severity of bacterial infections; thus, PCT can predict prognosis [18-20]. Normally, PCT does not increase when local bacterial infection occurs unless the infection is accompanied by systemic inflammatory reactions [20-22]. Most authors conclude that PCT has satisfactory diagnostic accuracy and an interesting clinical value for APN, with a sensitivity and a specificity ranging from 70% to 100% and 70% to 97%, respectively, across studies and thresholds [23-28]. However, a Belgium team found lower sensitivity and specificity (68% and 23%, respectively) with no obvious difference regarding the cutoff of the characteristics of the population [29]. The average PCT and CRP levels of children with APN were greatly higher than those of children with lower UTI (*P* < 0.01). In contrast, peripheral blood WBC counts were not significant in predicting renal involvement.

The areas under the PCT, CRP, and WBC curves were 0.958, 0.858, and 0.588, respectively, and the group analysis shows that the areas under both PCT and CRP curves displayed significant differences compared with those under the WBC curve. Both PCT and CRP can serve as laboratory indices for APN diagnosis, but PCT has a higher diagnostic value than CRP. The ROC curves in this study illustrate the same findings.

This study shows that PCR and CRP have a significant correlation with a Pearson’s correlation coefficient of 0.729 (*P* < 0.01). CRP also has a diagnostic value for APN diagnosis, but its sensitivity, specificity, and accuracy are low. The sensitivity and specificity of CRP, PCT, and WBC are related to a real positive patient’s threshold determination. Based on the results in this study, 1 ng/ml PCT can be considered the reference value because PCT has sensitivity of 90.47%, specificity of 88%, accuracy of 89%, a positive predictive value of 87%, and a negative predictive value of 95% in predicting APN.

The PCT and CRP levels after treatment significantly decreased compared with those before treatment (*P* < 0.05). Both PCT and CRP can be used for observing pathogenesis and curative effect. The serum PCT value in children with serious renal involvement was significantly higher than in those with mild and moderate renal involvement. A high PCT value indicates serious renal involvement. Therefore, PCT can be applied in predicting renal involvement. The CRP values in children with serious renal involvement were higher than those in children with mild and moderate renal involvement, but no significant difference was observed.

Serum PCT determination is an easy and cheap method for diagnosing APN, and only a small amount of blood is required. Furthermore, PCT is highly stable in serum, and the entire PCT determination process can be completed in 2 h. PCT determination can also be used for the observing curative effect and follow-up pathogenic condition sequelae, prognostic judgment, and renal involvement degree prediction. Serum PCT determination can be used in clinical settings.

The following are the limitations of this study. First, the sample size used was small. Second, PCT was initially measured with a quantitative immunoluminometric assay, but this assay was progressively replaced by PCT-sensitive KRYPTOR. Third, validation studies, threshold analyses, and studies on various effects are required before PCT is deemed safe for daily use.

## Competing interests

I declare that we have no financial competing interests.

## Authors’ contributions

R-YX participated in the design of the study, statistical analysis and drafting the manuscript. H-WL helped to carry out the immunoassays and data analysis. J-LL helped collecting blood samples. J-HD has given medical instruction. All authors read and approved the final manuscript.

## Pre-publication history

The pre-publication history for this paper can be accessed here:

http://www.biomedcentral.com/1471-2490/14/45/prepub
